# Successful Maternal‐Assisted Cesarean Using a Modified Australian Protocol

**DOI:** 10.1155/crog/7684108

**Published:** 2026-04-09

**Authors:** Caroline Lamoutte, Adaeze Anamege, Lauren Silva, Joanne J. Tanner, Natalie Elphinstone, Adam L. Wendling, Adetola F. Louis-Jacques

**Affiliations:** ^1^ Department of Obstetrics, Gynecology and Women’s Health, St. Louis University School of Medicine, St. Louis, Missouri, USA, slu.edu; ^2^ Department of Epidemiology, University of Florida College of Public Health and Health Professions and College of Medicine, Gainesville, Florida, USA, ufl.edu; ^3^ Department of Obstetrics and Gynecology, University of Florida College of Medicine, Gainesville, Florida, USA, ufl.edu; ^4^ Labor and Delivery Unit, University of Florida Health Shands Hospital, Gainesville, Florida, USA; ^5^ Department of Women’s Health, Peninsula Health, Frankston, Australia, peninsulacommunityhealth.co.uk; ^6^ Department of Anesthesiology, University of Florida College of Medicine, Gainesville, Florida, USA, ufl.edu

**Keywords:** maternal participation, operative obstetrics, patient-centered cesarean

## Abstract

**Background:**

Several delivery methods such as the natural, gentle, Charité, and family‐centered cesareans have been introduced to labor and delivery units to help facilitate immediate skin‐to‐skin, earlier breastfeeding, and bonding, as well as increase patient satisfaction with the birthing experience. For patients who deliver by cesarean and desire further involvement in the delivery process, maternal‐assisted cesarean (MAC) may be an option.

**Case Presentation:**

This case details a MAC at 39 weeks gestational age (GA) for a 31‐year‐old female with a history of two unplanned cesareans. The first was due to arrest of dilation and nonreassuring fetal heart tracing, and the second followed a failed trial of labor. In this current case, the patient desired a controlled delivery she could actively participate in. Following multidisciplinary planning and simulation, the cesarean was performed with modifications to maintain sterility while allowing maternal participation. After delivery of the fetal head and shoulders, sterile drapes were lowered, and the patient, guided by the surgical team, assisted in delivering the newborn onto her chest. Both mother and newborn remained stable postoperatively.

**Conclusion:**

This emerging patient‐centered cesarean enables active participation of the patient during delivery while maintaining surgical safety. Despite its rising popularity, studies are needed to evaluate its risks and benefits before wider adoption.

## 1. Introduction

While vaginal delivery is often preferred to cesarean delivery among birthing individuals, cesareans are widely accepted as safe and sometimes life‐saving operations for both the patient and baby. Over the last 50 years, cesarean rates in the United States (US) have increased substantially, comprising 5.5% of deliveries in 1970–31.8% in 2020 [1, 2], and make up about 20% of all deliveries globally [3]. While cesarean rates have remained relatively stable through the last decade in the US (32.9% in 2009 and 32.2% in 2022) [4, 5], this mode of delivery may be complicated, less desired by patients, and is associated with decreased patient satisfaction with the birthing experience when compared to vaginal birth [6]. Consequently, efforts have been made since the turn of the century to advocate for more patient‐centered practices in the obstetric operating room (OR). Such efforts have been named natural, gentle, Charité, and family‐centered cesareans, each aiming to engage parents in the birth of their babies beyond what has been traditionally practiced in the OR. For example, they integrate direct visualization of the delivery [7–9] and early skin‐to‐skin and breastfeeding [7–10] while still in the OR.

Another patient‐centered cesarean technique is the maternal‐assisted cesarean (MAC). In this method, the patient is intimately engaged in the delivery of the child with the aid of the surgeons [11]. To date, MAC has been sparsely described in peer‐reviewed literature, and any information available is predominantly from news sources, magazine articles, or blogs [12–17]. These mainstream sources are written with individuals of childbearing age as the target audience, empowering them to discuss birthing options available beyond traditional cesarean delivery [12–16]. To the authors’ knowledge, our institution is one of the first in the US to perform a MAC at a patient’s request [16, 17], although MAC has been practiced in Australia as far back as 2005 and continues to be used today [14, 18].

Here, we present the MAC case successfully performed at our institution along with an in‐depth description of the protocol used. The authors aim to increase awareness about MAC by utilizing a protocol that has been modified from an Australian institution and used at our hospital. We emphasize that this technique is likely best suited for routine cesarean deliveries of term fetuses in cephalic position in which maternal and fetal complications are unlikely to develop intrapartum.

## 2. Case Presentation

We describe the case of a 31‐year‐old G3 P2002 with a past surgical history significant for two cesareans who presented to our institution at 32 weeks gestational age (GA). The first cesarean under spinal anesthesia was due to arrest of dilation and nonreassuring fetal heart tracing remote from delivery, complicated by chorioamnionitis at 41 weeks GA. The second was a failed trial of labor after cesarean that culminated in an urgent cesarean under general anesthesia due to nonreassuring fetal heart tracing at 40 weeks 5 days GA. At the time of the scheduled MAC at 39 weeks and 2 days of gestation, the patient had no active obstetric complications. Her medical history was notable for a pregravid body mass index of 35 kg/m^2^ and a rhesus negative status. Written informed consent for publication was obtained. Consent was also obtained for all accompanying images.

This patient had always deeply desired a vaginal birth but, after the previous two unplanned cesareans, was hoping for a more controlled environment in which she and her partner could enjoy the delivery, not feel detached, and hopefully play a more active role in the delivery of their child than previously. Early in this pregnancy, she sought out information on patient‐centered cesareans and eventually discovered the MAC performed in Australia via social media. She was drawn to the idea of being able to participate in delivering her child with the surgeon, which, for her, was a clear and attractive parallel to the vaginal delivery she always desired. She began calling birthing centers and midwives to see if it was a possibility in her area and was eventually referred to our institution to make her request.

In the authors’ experience, MAC was initially proposed upon patient‐driven request and subsequently researched and discussed among key stakeholders such as the department perinatologists, obstetric nursing staff, and the anesthesia team. The first thing that needed to be determined was whether this patient was a suitable candidate for a MAC. The patient was counseled on other feasible options such as a trial of labor after cesarean and the other patient‐centered approaches [7–10]. MAC was still desired, and the patient was an appropriate candidate. The patient was amenable to proceeding with a traditional or another feasible patient‐centered cesarean should maternal‐infant safety become a concern. The patient and support person received educational materials through which they could familiarize themselves with routine procedures during the cesarean. Moreover, an interdisciplinary approach to the procedure was imperative. Communication between the nursing, anesthesiology, pediatric, and obstetric teams was established early in the planning process.

Once all key stakeholders agreed to proceed with the MAC, a walk‐through simulation with all indicated teams was performed prior to the day of delivery. The patient was also brought to the preoperative holding area and OR before the delivery date for a similar simulation. At this time, the patient was taught to perform a surgical scrub and was gowned and gloved while laying down, thereby simulating the procedure.

On the day of the surgery, the patient was accompanied to perform a wet scrub prior to routine nursing and anesthesiology assessments. Given that the patient was to be gowned and gloved for the cesarean, an intravenous line was placed high in the nondominant upper arm to make it easily accessible to the anesthesiology team and allow the patient maximum mobility of the dominant arm during delivery. The blood pressure cuff was placed on the dominant arm, and an ear pulse oximeter was used. Electrocardiogram leads were placed more peripherally on the patient’s chest, extremities, and back to allow easier access for direct skin‐to‐skin.

Once back in the OR, she was positioned for neuraxial anesthesia in the routine fashion, and continuous electronic fetal monitoring was utilized during placement. One concern was the incidence of nausea and vomiting after establishing neuraxial anesthesia, which could eventually compromise maternal sterility after the patient is gowned, thereby hindering the ability to proceed with the MAC delivery. Prior to spinal anesthesia, dual nonsedating antiemetic agents consisting of metoclopramide and ondansetron were utilized to reduce the likelihood of intraoperative nausea and vomiting [19, 20].

Once adequate regional anesthesia was established, the patient was placed in a supine position. The Foley, safety strap, sequential compression devices, and forced air warmer were placed, and fetal monitoring was discontinued. Tegaderm dressings were placed to fully cover the intravenous site prior to the patient’s dry scrub. The patient was then assisted by nursing staff in applying surgical scrub to their hands and forearms and was gowned and gloved in sterile fashion by the surgical technician. Arm boards were covered by sterile drapes, and the patient was instructed to keep her arms at breast level while her gown was folded upwards to expose the abdomen. The abdomen was subsequently surgically prepped in the routine fashion, and a sterile half‐sheet drape was clipped to two intravenous polls, creating a barrier for the sterile field. The patient’s support person was then escorted to the OR with a reminder to not touch the patient or the sterile field. The patient’s support person can also scrub and be gowned in a sterile fashion if the patient, surgical, and anesthesia teams agree to this modification.

**Figure 1:**
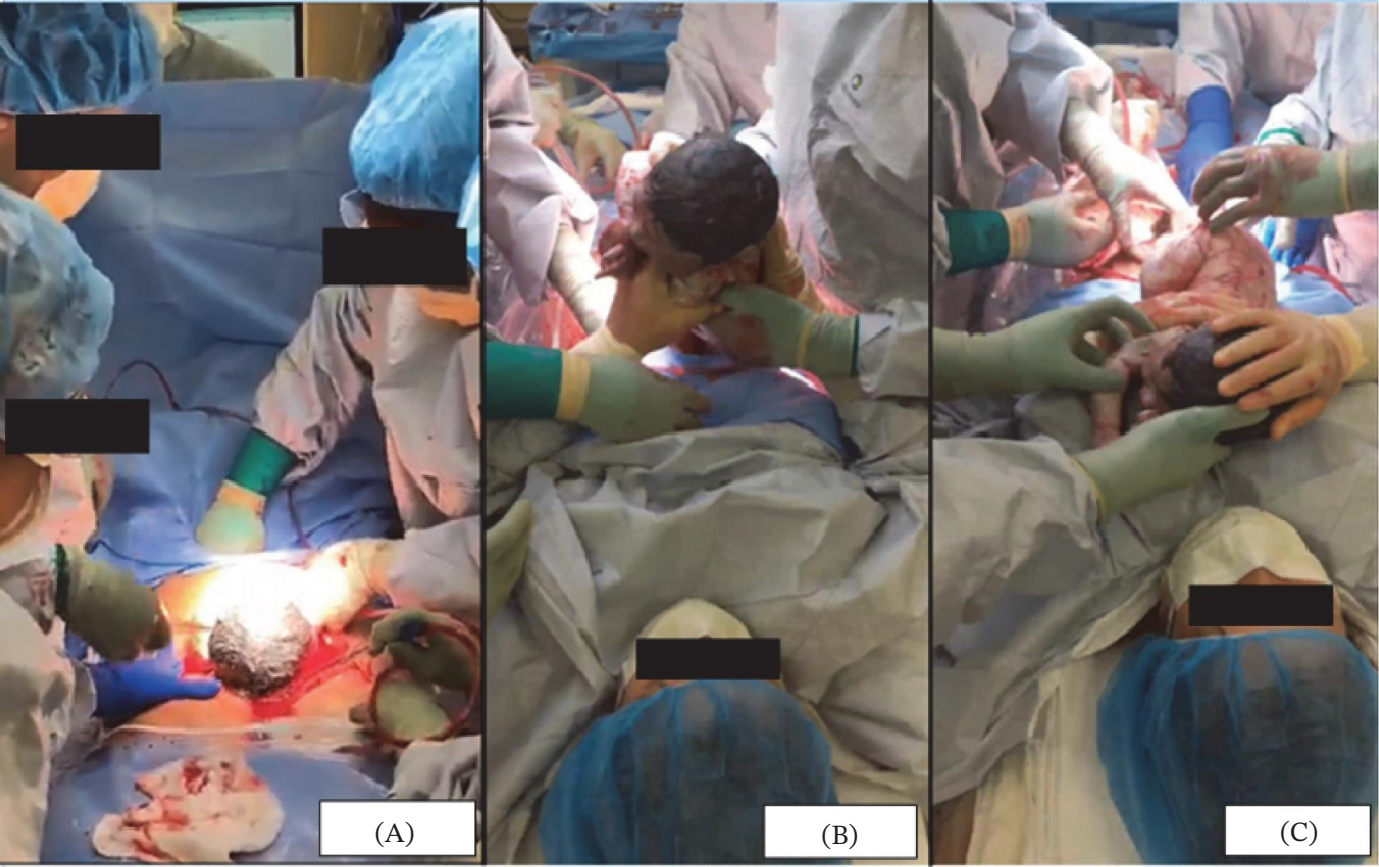
Ongoing maternal‐assisted cesarean showing the delivery of the baby’s head to initiate the hand‐over process (A), after which the surgical team is seen guiding the patient’s hands from the surgical site to her chest (B). The patient successfully receives and places the baby on her chest (C).

The cesarean proceeded with standard technique. Once the surgeons delivered the neonate’s head and shoulders (Figure 1A), the sterile drape barrier was lowered. The patient’s hands were guided by the surgeons to reach down, deliver the rest of the neonate, and pull the newborn to her chest (Figure 1B,C). Cord clamping was delayed for 60 s, and the patient was assisted in initiating skin‐to‐skin and breastfeeding.

A new sterile drape was then placed over the patient to protect the sterile field, and the remainder of the surgery was completed. Prophylactic oxytocin was provided for prevention of postpartum hemorrhage. In our case, the uterus was exteriorized for uterine closure and bilateral tubal ligation, and our patient tolerated it well. However, intra‐abdominal closure would have been considered if bilateral tubal ligation was not needed and uterine exteriorization was stimulating and emetogenic. Since the newborn remained stable, all neonatal assessments were performed on the patient’s chest. The quantitative blood loss was 1205 mL, and they were transferred together from the OR to the postanesthesia care unit in stable condition.

## 3. Discussion

Since the turn of the century, efforts have been made to advocate for more patient‐centered practices in the labor and delivery unit. Cesarean methods such as the natural, gentle, Charité, and family‐centered cesareans allow parents to observe the delivery of their newborn and help simulate the early mother–infant bonding that is commonly experienced during a routine vaginal delivery [7–10]. However, some patients may desire more direct involvement in the birthing process. MAC is unique in that it affords patients the opportunity to actively engage in the delivery process and allows participation in routine postvaginal delivery protocols.

As MAC becomes more publicized via mainstream media, we anticipate that patient requests for this technique may increase. However, successful implementation requires multidisciplinary planning across medical teams. Before a patient is scheduled to undergo a MAC, a multidisciplinary meeting including the participating obstetric, anesthesia, pediatric, and nursing teams should be arranged to determine whether the patient is an appropriate candidate, the proper staffing is available, and all providers are comfortable with the proposed procedural modifications. At our institution, the authors were impressed with each team’s leadership and willingness to try the method, so long as patient safety was maintained. Additionally, patient counseling and education are essential, particularly to highlight the importance of sterility.

Maintaining sterility throughout the procedure requires cooperation from all provider teams in the OR, as well as the patient and their support person. Because no studies have been performed on this method, it is not known whether this technique increases the risk for perioperative infection. This uncertainty should be communicated with the patient during the counseling process.

It is important to note that MAC may not be appropriate for all patients. This surgical technique is inherently more time‐consuming, both preoperatively and intraoperatively. Therefore, MAC deliveries may not be appropriate in emergency situations. There are also some clinical scenarios, such as breech presentation or fetal anomalies, that would make this method more challenging or less ideal. A modified approach in which the patient receives the child directly from the surgeons may be a possible alternative.

In an attempt to consolidate the limited data on patient‐centered cesareans, a recent systematic review and meta‐analysis was performed to compare these techniques—including the four mentioned earlier as well as early skin‐to‐skin in the OR—to traditional cesareans. The authors found no differences in maternal outcomes and most newborn outcomes with superior satisfaction and faster initiation of skin‐to‐skin and breastfeeding between the two groups [21]. These patient‐centered cesareans should be discussed with patients requesting MAC given that they are legitimate alternatives that have been shown to be beneficial. However, if a patient still desires a more direct involvement in delivery, MAC could be considered.

Cesareans are more often associated with a negative birthing experience when compared to vaginal deliveries [6]. MAC may help reduce such dissatisfaction and mitigate potential negative maternal‐neonatal outcomes associated with routine cesareans, including increased rates of postpartum depression [6]. Improving birthing experience is one approach to reduce postpartum depression rates, which is essential given that suicide is one of the leading causes of maternal mortality within the first 12 months postpartum [22].

Cesarean deliveries have also been associated with decreased breastfeeding rates, an association that was found to be no longer significant after adjusting for early breastfeeding initiation in one multiinstitutional study [23]. The MAC incorporates this early breastfeeding and skin‐to‐skin initiation, which has been shown to further enhance patient birthing experience [24] and function as a protective factor for newborns [25].

## 4. Conclusion

Patient satisfaction with the birthing experience, initiation of early breastfeeding, skin‐to‐skin, and maternal‐infant bonding play an important role in maternal and neonatal physical, social, and emotional health. Therefore, efforts should continue towards helping patients improve operative birthing experiences. Studies are needed to assess the risks and benefits of MAC.

## Author Contributions

Caroline Lamoutte is the primary author who performed a literature review and drafted the manuscript. Joanne J. Tanner wrote up the original protocol based on one from an Australian institution. Lauren Silva, Natalie Elphinstone, Adam L. Wendling, and Adaeze Anamege revised the manuscript critically, and Adetola F. Louis‐Jacques contributed to the manuscript concept and also revised the manuscript.

## Funding

No funding was received.

## Disclosure

All authors have read and approved the final version of the manuscript.

## Ethics Statement

This study is exempt from ethical approval as determined by the University of Florida Institutional Review Board.

## Consent

Written informed consent was obtained from the patient for publication of this case report and accompanying images.

## Conflicts of Interest

The authors declare no conflicts of interest.

## Data Availability

Data sharing is not applicable to this article, as no datasets were generated or analyzed during the current study.
